# Age and immunity

**DOI:** 10.1186/1742-4933-3-2

**Published:** 2006-02-24

**Authors:** Sonya Vasto, Marco Malavolta, Graham Pawelec

**Affiliations:** 1 Dipartimento di Biopatologia e Metodologie Biomediche, Università di Palermo, Italia; 2 Immunology Ctr.(Sect.: Nutrition, Immunity and Ageing)Res. Dept. INRCA, Ancona Italia; 3 Center for Medical Research, University of Tübingen, D-72072 Tübingen, Germany

## Abstract

Longitudinal studies are defining progressive alterations to the immune system associated with increased mortality in the very elderly. Many of these changes are exacerbated by or even caused by chronic T cell stimulation by persistent antigen, particularly from Cytomegalovirus. The composition of T cell subsets, their functional integrity and representation in the repertoire are all markedly influenced by age and by CMV. How these findings relate to epidemiological, functional, genetic, genomic and proteomic studies of human T cell immunosenescence was the subject of intense debate at an international conference held just before Christmas 2005 in the Black Forest.

## Background

This conference was the final meeting of the EU project "T-Cells in Aging", T-CIA [1]. T-CIA brought 8 European centers together for 3 years to study T cell immunosenescence in clonal models *in vitro *and *ex vivo *in the elderly. The former focused on long-term cultures of T cell clones (TCC), the latter on longitudinal studies of the very elderly. This work formed the core of the data presented at the meeting in Freudenstadt-Lauterbad, 13–16 December, 2005. The T-CIA project, coordinated by **Graham Pawelec **(Tübingen University) evolved from the earlier EU-supported networking projects EUCAMBIS and ImAginE, and has investigated human T-cell immunosenescence in vivo end in vitro with the aim of increasing the understanding of immune ageing and laying the preclinical groundwork for rational intervention.

Senescence of clonotypic immunity is thought to be principally a result of the declining effectiveness of T cells. Lifelong exposure to chronic antigenic load is the major driving force of immunosenescence, impacting on human lifespan by reducing the number of naïve antigen-non-experienced T cells, and, simultaneously, filling the immunological space with expanded clones of memory and effector, antigen-experienced T cells [2]. Gradually, the T-cell population shifts to a lower ratio of naïve to memory cells, the thymus generates fewer naïve T cells with age and those T cells remaining, especially the CD8+ subset, also show increased oligoclonality with age. Thus, the repertoire of cells available to respond to antigenic challenge from previously unencountered pathogens is shrinking. In addition, older individuals commonly possess memory cells carrying a single T cell receptor, i.e. they represent clonal expansions. Thus, the memory cells from old individuals might recognize a limited set of antigens despite being plentiful in number. Many of the clonal expansions crowding an elderly person's immune system result from previous infections by persistent viruses, resulting in the presence of many CD8+ cells specific for a limited number of epitopes of herpes viruses: in some individuals more than 10% of peripheral CD8 cells react against a single CMV epitope [3]. Here, then, there is potential for intervention, both prophylactic (vaccination), therapeutic (anti-virals) and immunologic (deletion of dysfunctional cells). A novel approach to the latter was suggested by **Katsuiku Hirokawa (Tokyo Medical & Dental University) **who proposed banking peripheral T cells from younger individuals, depleting their dysfunctional cells in later life, and replenishing from the frozen bank of young T cells. This type of "mini-transplant" is standard procedure in cancer therapy. Memory T cells usually carry the CD28 surface protein, which helps stimulate the cells to divide when antigen is present. But old memory cells tend to lose CD28 and, as a result, multiply less robustly when exposed to antigen than do younger cells [3]. Here, therefore, is another opportunity for intervention: cause re-upregulation of CD28, either by gene therapy or application of cytokines such as interleukin (IL)-12 or antibodies neutralising tumor necrosis factor (TNF)-α. As a consequence of the above events, if unchecked, T and B immunosenescence and, likely, mortality and morbidity, will occur earlier in people that have been exposed to an antigenic overload (due to chronic infections). The opposite situation may apply to people exposed to a lower antigenic load and/or with an appropriate immunogenetic background. This implies that the ability of our immune system is progressively worn down by the need to maintain immunosurveillance against persistent pathogens or other sources of antigen, such as cancer [4].

### Cytomegalovirus (CMV)

For the reasons discussed above, a good part of the meeting, like the elderly immune system, was obsessed with CMV. **Paul Moss (University of Birmingham) **showed that cytomegalovirus infection at any stage in life adds more age to the ageing process with respect to the naïve compartment. Indeed, the immune response to CMV can account for around 25% of all the clonal CD8+ T cell populations that are known to accumulate with ageing. Concomitantly, the absolute T cell count is increased by 20% in elderly donors who are CMV-positive compared with CMV sero-negative. This correlates with the absolute count of CD8+ T cells doubling in the CMV-seropositive cohort and with the CD8+CD28- subset dramatically increased 5-fold. Because CMV infection results in such strong T cell responses, methods to control infection and to prevent reactivation through vaccination might be beneficial [5]. According to the old adage "know your enemy" maximising our information on CMV-immune system interactions might also facilitate control of the beast. **Stefan Stevanovic (University of Tübingen**) investigated CMV-specific T cell responses in a large number of healthy donors of different human leukocyte antigen (HLA) types and found that as previously reported, almost all HLA-A*01-, A*02-, and B*07- positive individuals had TCR specific for a limkited range of immunodominant epitopes. However, people with other HLA-allotypes did not mediate such immunodominant responses, suggesting that control by vaccination with a limited number of epitopes may be more complicated [6].

In the elderly, **Rafael Solana (University of Cordoba**) observed higher percentages of CMV-specific CD8 effector memory cells, and showed that it was these cells which predominantly expressed enhanced levels of HLA class-I-specific and non-specific NK receptors [7]. These results suggest that interactions usually occurring within the innate immune system will need to be taken into count when considering CMV control strategies. **Paolo Sansoni (University of Parma**) confirmed that age-associated CD8+ T clonal expansion could be related to increased frequency of CMV infection. In particular, CD28- T cells accumulate, with age, only in seropositive subjects both within CD4+ and CD8+ cells while reduction of naïve T cells is more profound among CMV-seropositive subjects within CD8+ T cells. He also found that IFN-γ -producing cells specific for at least one immunodominant CMV protein within CD8+ or CD4+ subsets increase with age [8]. These results indicate that while attention has focussed on the larger clonal expansions seen in CD8 cells, effects on CD4 cells are also likely to be critical in shaping the elderly immune response.

## Apoptosis

Aging is associated with a paradox of increased autoimmunity, yet also with a state of immunodeficiency, with an increased frequency of infections and cancer. Among immune functions, a decline in T-cell functions during aging predominates. In aging, there is an increased production of TNF-α and decreased naive CD8+ T cells whereas memory CD8+T cells are increased, under the influence of CMV as discussed above. **Sudhir Gupta (University of California) **reported that naïve and central memory (TCM) CD8+ T cells are decreased in ageing, whereas the CD45RA- effector memory (TEM) and CD45RA+ effector memory (TEMRA) CD8+ T cells are increased. The hypothesis is that the alterations in naïve and CD8+T memory cells in ageing may be due to their differential sensitivity to apoptosis. Naive and TCM CD8+T cells from old donors are significantly more susceptible to TNF-α-induced apoptosis as compared to naïve and TCM from young subjects, whereas TEM and TEMRA CD8+ T cells are resistant to apoptosis in both young and aged subjects. Furthermore, in ageing, apoptosis in TCM CD8+ T cells, as defined by CCR7 or CD62L expression, was greater as compared to naive CD8+ T cells. It was also observed that the increased apoptosis in aged naive and TCM CD8+ T cells was associated with increased cleavage of both caspase-8 and caspase-3 as compared to young subjects [9,10]. This kind of increased apoptosis of naïve and central memory T cells may contribute to T cell immunodeficiency associated with human ageing. In contrast, abnormal phagocytosis of apoptotic body and failure to generate anti-inflammatory response by dendritic cells may explain the paradoxical increased inflammation associated with human ageing. Modulation of these processes may therefore also offer scope for intervention.

## Genetics

The pathogenic burden, to which an individual has been or is exposed, may be linked to levels of chronic inflammation and to increased risk of age-related diseases. This will be influenced by genetic factors affecting the capacity of the host to control the pathogen-induced inflammatory response. **Calogero Caruso (University of Palermo) **suggested that within the inflammatory network, the most important node of the net is represented by CD14, the main receptor for Gram-negative endotoxin, and its co-receptor, TLR4, responsible for activating intracellular signalling pathways. Several functional polymorphisms have been described in these genes associated with age-related disease. Thus, the intensity of the genetically-determined inflammatory response against pathogens or their antigens might play a major role in determining the magnitude of inflammation and subsequent clinical outcome. The presence of gene polymorphisms with pro-inflammatory associations may fuel the inflammatory response of macrophages to gram-negative infection, promoting pro-inflammatory status and clinical setting of the inflammatory diseases [11,12]. This implies that people genetically predisposed to a weak inflammatory activity have less risk of developing age-related diseases and genetic polymorphisms responsible for a low inflammatory response might therefore result in an increased chance of a long life-span in a modern environment with a reduced pathogen burden.

**Elisavetta Naumova (University of Sofia) **studying the Bulgarian population focused on the relevance of HLA and cytokine genotype profiling as predictors of successful ageing. The analysis showed statistically significantly increased frequencies in elderly individuals and in families with long-lived members of HLA haplotypes such as DRB*11 and DRB*16. However, the pro-inflammatory cytokine gene polymorphisms for IL-2, IL-6, IFN-γ did not differ significantly between elderly and controls, but differences were observed for IL-10, TGF-β, and TNF-α genes, supporting a role of HLA and cytokine genes in successful ageing [13].

Another factor with a genetic basis discussed in this session, which could be contributing to T cell immunosenescence and chronic inflammation in the elderly, is the dysregulation of zinc homeostasis **(Marco Malavolta, INRCA, Ancona). **Preliminary results from the EU ZINCAGE Project, indicate that zinc dyshomeostasis, characterized by high levels of expression of metallothioneins (MT) associated with plasma zinc deficiency and impaired release of zinc at the intracellular level, may occur mostly in aged T cells of subjects carrying the C allele of the 647 A/C MT1A SNP, especially when associated with a pro-inflammatory cytokine profile. In contrast, carriers of the A allele, perhaps through a better regulation of zinc homeostasis, have a greater likelihood of longer life [14]. Careful zinc supplementation my therefore also represent a possible intervention to help maintain immunological integrity, an approach that the ZINCAGE project is currently testing.

**Daniel Remondini (Bologna University) **presented data on T-cell profiling over lifespan in humans, suggesting a possible global response of gene activity in ageing.

## In vivo correlates

Although the immune system exhibits profound age-related changes, it has been suggested that old individuals frequently do retain some capacity to produce fully functional new T cells. However, how we can define naïve cells in the elderly? This question [15] was addressed by **Beatrix Grubeck-Loebenstein (Institute of Biomedical Ageing Research, Innsbruck)**, who reported her quest for truly naïve cells in the elderly within a theoretically naïve population (as defined by co-expression of the surface moleculesCD8+ CD28+ CD45RA+ CD62L+). What emerged is that these "naïve cells" had shorter telomeres and a more limited antigen receptor repertoire in the old than in the young. This implies that "naïve cells" in the elderly have undergone considerable division and may be considered "aged cells" despite being phenotypically naïve. It also raises the question as to whether these markers of "naïve" cells are reliable.

It is becoming clear that external factors like infections occurring early in life may affect T cell functions in the elderly, first and foremost, CMV infection. There are, however, also non-infectious diseases recognized as associated with impaired functionality of CD4+ T cells and known to significantly shorten life span. Among them, **Jacek M. Witkowski (Medical University of Gdansk) **suggested that rheumatoid arthritis (RA) is associated with accelerated ageing of CD4+ T cells. Thus, physiological ageing- and RA-associated characteristics of CD4+ lymphocytes display marked similarities and include identical patterns of change of dynamic parameters of proliferation stemming from similar underlying mechanisms, like increase expression of cyclin D1 and decreased expression of the CD28 co-stimulatory molecule. Also in two other "life-shortening" diseases, systemic lupus erythematosus and Alzheimer disease, the CD4+ cells are primed for activation, showing a decreased duration of the G0→G1 transition time, yet associated with a reduced ability to produce viable progeny [16].

Longitudinal studies in human populations are financially and logistically challenging, but yield uniquely informative data. The OCTO/NONA studies described by **Anders Wikby (Jönköping University) **resulted in the development of the concept of an "Immune Risk Profile "(IRP) in population-based samples of individuals aged 86–99 years. The factors studied now include analysis of T cell subsets, inflammatory markers, virus serology, cytokines, DNA damage, TCR clonotype mapping, and functional and phenotypic analysis of virus-specific CD8+ cells by HLA/peptide multimers. These studies emphasize the paramount importance of multi-disciplinary collaboration between many different laboratories specialised in different areas, as illustrated by the T-CIA project [17].

## Mechanism

The elderly possess more or less the same numbers of peripheral T cells as the young, but as we have seen above, of very different subset and clonal composition. In addition, age-associated alterations on a per-cell basis contribute to immunosenescence, as illustrated by altered signal transduction pathways and processes in cells from old donors. **Tamas Fulop (University of Sherbrooke, Canada) **gave a general overview on the mechanisms of signal transduction through the T cell receptor, remarking on the key role played by lipid rafts in the formation of the initial complex of signal transduction for T-cell activation. Age-related changes in the cell membrane and specifically impacting on lipid rafts, (cholesterol content, fluidity and signalling molecule composition) may help to explain the severe impairment of CD4+ T-cell signalling observed in ageing, taking also into account that CD4+ T-cell activation completely relies on of lipid raft polarization. Furthermore, a different signalling of the CD28 co-receptor has been shown for the first time among CD4+ and CD8+ T cells from elderly subjects, independent of the measurable CD28 receptor number [19]. This suggests that the physico-chemical properties of the membrane influences more the signalling of a receptor thanthe receptor number *per se*." [18-20]. In stark contrast, and clearly something that must always be considered when thinking about the emerging differences between CD4 and CD8 cells, activation of the latter is independent of lipid raft polarization. Along the same lines, **Anis Larbi (University of Tubigen) **compared membrane fluidity and cholesterol content, two key parameters in lipid raft function and consequently TCR signalling, in T cell clones (TCC) derived from CD34+ progenitor cells, young adult donors or centenarians. Cholesterol content decreased while membrane fluidity increased during "in vitro" ageing, but stimulation via the TCR and CD28 led to different phosphorylation patterns depending more on the age of the donor than the in vitro age of the clones. These results possibly suggest that TCC may represent intrinsically divergent properties of the donor cells rather than culture-induced changes [18]. However, they also show that intervention using lipids, either injected or ingested, may result in some degree of immune modulation of potential benefit to the elderly. Further studies on signal transduction, as related to telomerase expression in CD8+ T cells, were presented by **Fiona Plunkett (University College, London). **Investigating telomere length, telomerase expression, proliferative capacity, and co-stimulatory receptor expression in the various CD8+ CD28/CD27 subpopulations, she showed that CD8+CD28-CD27- T cells have short telomeres and a decreased capacity to up-regulate telomerase in the absence of exogenous cytokines such as IL-2 and IL-15. Using chimeric receptors, it was also shown that up-regulation of telomerase expression in CD8+ T cells can be achieved through other co-stimulatory molecules, such as CD27, CD137 and ICOS, and not only via CD28. This suggests that CD8+CD28-CD27- T cells could still function if given appropriate alternative costimulation and a conducive cytokine milieu for their full activation [21].

Not only does T-helper and cytototoxic cell activation at the single cell level change with ageing, but the frequency and behaviour of regulatory T cells is attracting increased attention once more, also in the context of ageing. The maintenance of immunity has to be balanced with appropriate controls to prevent non-specific inflammation and immunopathology, which it is thought are major problems in ageing. **Arne Akbar (University College, London)**, studying the susceptibility to apoptosis, telomere length, turnover and clonal composition of the regulatory population, reported that CD4+CD25+ T-regulatory cells (T-regs) are generated continuously, most likely by differentiation of CD4+ T-cells in the presence of regulatory cues [23]. These concepts on the mechanisms and dynamic contributing to peripheral tolerance by CD4+CD25+ T-regs, have important implications for the design of therapeutic strategies involving generation and use of CD4CD25+ T-regs in autoimmune and inflammatory diseases. Clearly, their manipulation in ageing will also be of great potential benefit.

Given the difficulty of finding (or defining) naïve cells in the elderly, and given the well-know phenomenon of thymic involution, one focus of attention for researchers interested in immunosenescence must be the thymus itself. As discussed by **Richard Aspinall (Imperial College, London) **interventionist therapies, based around IL-7, aimed at rejuvenating the thymus to the size and cellularity seen during early life and restoring thymic output, may be an attractive avenue to explore. In particular, treatment with a CCR9/IL-7 fusion protein, which retains its IL-7 activity, seems particularly intriguing because it displays an increased ability to target only the thymus, thus avoiding any side effects of high-dose IL 7 (which may be lymphomagenic). In fact, mice receiving this fusion protein responded better to influenza infection (in terms of CD8 activation, viral load in the lung and change in weight) than IL-7 or untreated animals [22].

Even given the possibility of enhancing naïve T cell generation and improving signal transduction for activation, genomic instability and repair mechanisms of vigorously proliferating cells remains a major concern. **Alexander Burkle (University of Constance)**, reviewed the role of poly(ADP-ribosyl)ation and poly(ADP-ribose) polymerase-1 (PARP-1) in retarding the accumulation of DNA damage and in slowing down the rate of ageing [24]. Molecular genetic approaches to modulate poly(ADP-rybosyl)ation and an innovative method to assess poly(ADP-ribosyl)ation capacity by flow cytometry will facilitate study of and intervention in this critical gatekeeping system. Repair mechanisms in T cells were also addressed by **Erminia Mariani and Simona Neri (University of Bologna)**, who investigated the mismatch repair system (MMR) in T CC derived from hematopoietic stem cells and peripheral T cells from young, old and centenarian donors. By analysing mutations in particular non-coding regions (microsatellites), they observed that in vitro replication increased genomic instability and altered MMR gene expression especially markedly in CD34+ cell-derived clones. This can be seen also in TCC in relation to donor age, suggesting a reduced efficiency of the MMR system during in vitro ageing [25]. Improving DNA repair mechanisms would obviously be a general benefit, not only in ageing but also in cancer and other diseases.

The challenge to find key modulators of immunosenescence and ageing was addressed by **Dawn Mazzatti (Unilever R&D Colworth, UK) **combining genomic and proteomic investigations on T-lymphocytes isolated from young and old donors, as well as TCC grown to senescence in vitro. The proteins which were demonstrated to be differentially expressed in immunosenescence in TCC following SELDI analysis and further identified by MALDI/ESI-MS/MS were associated with SELDI peaks at 13–14kDa (identified as Histone H2B.q, H2A.5, H2A.q, H2B, and H2B.1) and 8.3–8.5kDa (Tetraubiquitin, chain B and Ubiquitin mutant). Genes found to be differentially expressed in human ex vivo samples during ageing belonged to variety of functional processes which were analysed by Ingenuity Pathway analysis software. These include metabolism, cytoskeleton remodelling, histone organisation, cell-cell communication, signalling and protein degradation. All these gene/proteins might constitute possible targets to ameliorate immunosenescence in the near future. In the meantime, a more direct approach with different antioxidants/ROS scavengers aimed at alleviating the dangerous effects of reactive oxygen species (ROS) on TCC was applied by **Yvonne Barnett (Nottingham Trent University, Nottingham)**. She found that carnosine and N-tert-butyl-α-phenylnitrone (PBN) extended the lifespan of TCC, at the same time as reducing DNA damage. Paradoxically, and contrary to expectations, a superoxide dismutase mimetic (EUK-134) and reduced oxygen tension (6%) culture conditions shortened the lifespan and proliferative capacity of TCCs, despite protecting against DNA damage [26]. These results suggests that better protection against free radicals might be useful to achieve functional longevity extension of human TCCs, but also that functional mechanisms requiring free radical production suggest complexity of applying anti-oxidant approaches in living cells.

## Conclusion

Improving our understanding of immune ageing is a necessary step towards identifying ways to ameliorate and treat the underlying causes of immunosenescence. Presently, there are very few treatments that seem both clinically applicable and efficacious, but some of the preclinical rationales for interventions discussed at this T-CIA final conference, some of which are mentioned above, may open promising perspectives. Application of most of the possible, but untested, interventions in the elderly is, however, fraught with ethical as well as medical difficulties in the case of people who are not overtly sick. These, as well as the scientific problems, now require discussion as a matter of priority.

## References

[B1] http://www.medizin.uni-tuebingen.de/t-cia/.

[B2] Ouyang Q, Wagner WM, Zheng W, Wikby A, Remarque EJ, Pawelec G (2004). Dysfunctional CMV-specific CD8(+) T cells accumulate in the elderly. Exp Gerontol.

[B3] Ouyang Q, Wagner WM, Wikby A, Walter S, Aubert G, Dodi AI, Travers P, Pawelec G (2003). Large numbers of dysfunctional CD8+ T lymphocytes bearing receptors for a single dominant CMV epitope in the very old. J Clin Immunol.

[B4] Walter S, Bioley G, Buhring HJ, Koch S, Wernet D, Zippelius A, Pawelec G, Romero P, Stevanovic S, Rammensee HG, Gouttefangeas C (2005). High frequencies of functionally impaired cytokeratin 18-specific CD8+ T cells in healthy HLA-A2+ donors. Eur J Immunol.

[B5] Khan N, Hislop A, Gudgeon N, Cobbold M, Khanna R, Nayak L, Rickinson AB, Moss PA (2004). Herpesvirus-specific CD8 T cell immunity in old age: cytomegalovirus impairs the response to a coresident EBV infection. J Immunol.

[B6] Nastke MD, Herrgen L, Walter S, Wernet D, Rammensee HG, Stevanovic S (2005). Major contribution of codominant CD8 and CD4 T cell epitopes to the human cytomegalovirus-specific T cell repertoire. Cell Mol Life Sci.

[B7] Pawelec G, Akbar A, Caruso C, Solana R, Grubeck-Loebenstein B, Wikby A (2005). Human immunosenescence: is it infectious?. Immunol Rev.

[B8] Vescovini R, Telera A, Fagnoni FF, Biasini C, Medici MC, Valcavi P, di Pede P, Lucchini G, Zanlari L, Passeri G, Zanni F, Chezzi C, Franceschi C, Sansoni P (2004). Different contribution of EBV and CMV infections in very long-term carriers to age-related alterations of CD8+ T cells. Exp Gerontol.

[B9] Gupta, Sudhir, Gollapudi, Sastry (2006). "TNF-a-induced apoptosis in human naive and memory CD8+ T cells in aged humans". Experimental Gerontology.

[B10] Gupta S (2005). Molecular mechanisms of apoptosis in the cells of the immune system in human ageing. Immunol Rev.

[B11] Caruso C, Candore G, Colonna-Romano G, Lio D, Franceschi C (2005). Inflammation and life-span. Science.

[B12] Balistreri CR, Candore G, Colonna-Romano G, Lio D, Caruso M, Hoffmann E, Franceschi C, Caruso C (2004). Role of Toll-like receptor 4 in acute myocardial infarction and longevity. JAMA.

[B13] Naumova E, Mihaylova A, Ivanova M, Michailova S, Penkova K, Baltadjieva D (2004). Immunological markers contributing to successful aging in Bulgarians. Exp Gerontol.

[B14] Mocchegiani E, Bertoni-Freddari C, Marcellini F, Malavolta M (2005). Brain, aging and neurodegeneration: role of zinc ion availability. Prog Neurobiol.

[B15] Pawelec G, Akbar A, Caruso C, Effros R, Grubeck-Loebenstein B, Wikby A (2004). Is immunosenescence infectious?. Trends Immunol.

[B16] Witkowski JM, Bryl E (2004). Paradoxical age-related cell cycle quickening of human CD4+ lymphocytes; a role for cyclin D1 and calpain. Experimental Gerontology.

[B17] Wikby A, Ferguson F, Forsey R, Thompson J, Strindhall J, Lofgren S, Nilsson BO, Ernerudh J, Pawelec G, Johansson B (2005). An immune risk phenotype, cognitive impairment, and survival in very late life: impact of allostatic load in Swedish octogenarian and nonagenarian humans. J Gerontol A Biol Sci Med Sci.

[B18] Fulop T, Larbi A, Dupuis G, Pawelec G (2003). Ageing, autoimmunity and arthritis: Perturbations of TCR signal transduction pathways with ageing – a biochemical paradigm for the ageing immune system. Arthritis Res Ther.

[B19] Larbi A, Dupuis G, Khalil A, Douziech N, Fortin C, Fulop T Differential role of lipid rafts in the functions of CD4(+) and CD8(+) human T lymphocytes with aging. Cell Signal.

[B20] Larbi A, Dupuis G, Douziech N, Khalil A, Fulop T (2004). Low-grade inflammation with aging has consequences for T-lymphocyte signaling. Ann N Y Acad Sci.

[B21] Plunkett FJ, Franzese O, Belaramani LL, Fletcher JM, Gilmour KC, Sharifi R, Khan N, Hislop AD, Cara A, Salmon M, Gaspar HB, Rustin MH, Webster D, Akbar AN (2005). The impact of telomere erosion on memory CD8+ T cells in patients with X-linked lymphoproliferative syndrome. Mech Ageing Dev.

[B22] Henson SM, Snelgrove R, Hussell T, Wells DJ, Aspinall R (2005). An IL-7 fusion protein that shows increased thymopoietic ability. J Immunol.

[B23] Taams LS, van Amelsfort JM, Tiemessen MM, Jacobs KM, de Jong EC, Akbar AN, Bijlsma JW, Lafeber FP (2005). Modulation of monocyte/macrophage function by human CD4+CD25+ regulatory T cells. Hum Immunol.

[B24] Burkle A, Diefenbach J, Brabeck C, Beneke S (2005). Ageing and PARP. Pharmacol Res.

[B25] Neri S, Cattini L, Facchini A, Pawelec G, Mariani E (2004). Microsatellite instability in in vitro ageing of T lymphocyte clones. Exp Gerontol.

[B26] Pawelec G, Mariani E, McLeod J, Ben-Yehuda A, Fulop T, Aringer M, Barnett Y (2004). Engineering anticancer T cells for extended functional longevity. Ann N Y Acad Sci.

